# The neutrophil-to-lymphocyte ratio and lactate dehydrogenase combined in predicting liver metastasis and prognosis of colorectal cancer

**DOI:** 10.3389/fmed.2023.1205897

**Published:** 2023-06-23

**Authors:** Qin Chen, Guo-lin Li, Hong-quan Zhu, Jian-Dong Yu, Zhi-Ping Chen, Jia-Yan Wu, Ze-Yu Lin, Yun-Le Wan

**Affiliations:** ^1^Department of general Surgery, The No.2 People’s Hospital, Wuxi, Jiangsu, China; ^2^Department of General Surgery (Hepatobiliary Surgery), The Sixth Affiliated Hospital, Sun Yat-sen University, Guangzhou, Guangdong, China; ^3^Biomedical Innovation Center, The Sixth Affiliated Hospital, Sun Yat-sen University, Guangzhou, Guangdong, China; ^4^Department of General Surgery, Jiangmen Central Hospital, Jiangmen, Guangdong, China

**Keywords:** neutrophil-to-lymphocyte ratio, lactate dehydrogenase, the combination of NLR and LDH (NLR-LDH), colorectal cancer liver metastasis, prognosis

## Abstract

**Background:**

The neutrophil-to-lymphocyte ratio (NLR) and lactate dehydrogenase (LDH) level are inflammatory markers related to tumor growth and metabolism. This study investigated the value of preoperative NLR, LDH and the combination of NLR and LDH (NLR-LDH) for predicting colorectal cancer liver metastasis (CRLM) and tumor prognosis in the early stages of colorectal cancer (CRC).

**Materials and methods:**

Three hundred patients undergoing CRC resection were included. Logistic regression analysis was used to estimate the correlation between CRLM time and inflammatory markers, and Kaplan–Meier survival and Cox regression analyses were used to estimate overall survival (OS). Forest plots were prepared based on the multivariate Cox analysis model and evaluated by receiver operating characteristic (ROC) curve analysis.

**Results:**

The NLR cut-off value was 2.071 according to the ROC curve. The multivariate analysis showed that the elevated LDH level and a high NLR-LDH level were independent predictors of synchronous CRLM and OS (*p* < 0.05). The combination of a high NLR and elevated LDH and NLR-LDH levels suggested a poor prognosis and a significantly shorter median survival time than a low NLR and low levels of LDH and NLR-LDH. The ROC curve analysis results illustrated that the predictive value of the NLR-LDH score for synchronous CRLM [area under the curve (AUC) = 0.623, *p* < 0.001] and OS (AUC = 0.614, *p* = 0.001) was superior to that of the NLR or LDH score used alone.

**Conclusion:**

LDH and NLR-LDH are reliable, easy-to-use, independent biomarkers for predicting synchronous or metachronous CRLM and OS in CRC patients. The NLR is an important monitoring index for CRLM. Preoperative NLR, LDH and NLR-LDH may help to guide the use of therapeutic strategies and cancer surveillance.

## Introduction

Colorectal cancer (CRC) is the third most common cancer in the world and the second leading cause of cancer-related death (1, 2). The liver is the most common site of CRC metastases and is a leading cause of death in patients with CRC. Radical surgery significantly improves the survival of patients with colorectal cancer with liver metastasis (CRLM) (3, 4). However, it is estimated that currently R0 resection occurs in only 10–20% of patients (5). Therefore, there is increasing need to develop reliable and easy-to-use biomarkers that can identify patient groups at high-risk of CRLM before treatment. Patient stratification based on these predictive biomarkers will allow individualized treatment in the early stage to improve the resection rate of LM.

Inflammation is considered as one of the major hallmarks of cancer (6, 7). As early as 1863, Rudolf Virchow linked inflammation to cancer (8). Studies have shown that inflammation-related cytokines can affect the growth and proliferation of tumor cells and that tumor cells themselves can secrete proinflammatory cytokines to regulate and communicate with each other cells (9). A growing body of clinical and experimental evidence also suggests that inflammation can promote tumor development and predict survival in a variety of cancers (10–12). In recent years, the neutrophil-to-lymphocyte ratio (NLR), as an important indicator of systemic inflammation, has been found to be closely related to patient survival and has been used as a biomarker for cancer prognosis (13, 14).

Inflammation also promotes glycolysis and the production of lactic acid (15). In fact, most cancer cells are chronically hypoxic in the early stages of carcinogenesis, and these cells rely on glycolysis as a source of ATP (16). Warburg found that even when there is enough oxygen to support mitochondrial oxidative phosphorylation, cancer cells are still predominantly supported by glycolysis, also known as aerobic glycolysis (17). During the growth of solid tumors, rapidly proliferating cells require more sustained energy to grow and survive (18, 19). To support this high metabolic demand, glycolysis is highly active in various tumor tissues (20, 21). Therefore, the status of glycolysis is considered to have potential value in predicting the prognosis of patients. In addition, lactate dehydrogenase (LDH), as a key enzyme in glycolysis, catalyzes the conversion between pyruvate and lactic acid and is closely related to tumor angiogenesis (22, 23), which may be a marker of the rapid cell metabolism of highly proliferative tumor cells (24).

Blood is a rich source of tumor-related biomarkers (25). The advantages of easy and non-invasive access to peripheral blood make inflammation-related peripheral blood factors, such as different blood counts (white blood cells, etc.), the ratio of blood counts (NLR, PLR, etc.) and lactate dehydrogenase (LDH), attractive potential biomarkers (26–29). However, there is lack of study on the ability of the combined peripheral blood inflammatory index NLR-LDH to predict the LM and prognosis of CRC. To our knowledge, our study is the first to combine peripheral inflammatory cytokines, NLR, LDH and NLR-LDH combined indexes to predict the time of CRLM and tumor prognosis in the early stage of CRC.

## Patients and methods

### Patients

All patients were identified as having CRC and their samples were provided anonymously for clinical study.

A total of 9,514 patients with CRC who underwent radical resection in the Sixth Affiliated Hospital, Sun Yat-sen University from December 2013 to August 2018 were screened. Patients meeting the following criteria were enrolled: (1) The patient was diagnosed with CRC by histopathology and underwent primary radical resection of CRC. (2) The patient developed synchronous or metachronous CRLM. (3) The number of lymph node biopsies was greater than 12. (4) The patient had no blood diseases, infectious diseases, immune diseases, or other diseases caused by poor nutritional status. (5) The patient had no intestinal perforation and/or severe ileus. (6) The patient did not receive drugs that may affect markers of inflammation. (7) Death occurred only from tumor-related causes. (8) The patient had complete clinical data of laboratory examination, imaging examination and follow-up.

### Data extraction and follow-up

Clinical data, including patient age at diagnosis, gender, location of primary tumor, histological differentiation, T stage, N stage, CRLM features and treatment regimens, were extracted from electronic medical records. The laboratory data, including neutrophils, lymphocytes, LDH, carcinoembryonic antigen (CEA), carbohydrate antigen 19–9 (CA19-9) and carbohydrate antigen-125 (CA-125), were collected from peripheral blood within 7 days before the surgical resection of their primary tumor. The pathological stage of CRC was classified according to the American Joint Committee on Cancer (AJCC) tumor-node-metastasis (TNM) staging system, eighth edition. All patients were followed up until August 2020 or until death. The primary endpoint was overall survival (OS).

### Endpoints and time frame of LM

OS was defined as the time interval from the date of diagnosis to death from any cause or to the last known date of life. Synchronous CRLM was defined as metastasis that occurred at the time of diagnosis of CRC or within 6 months after radical resection of the primary CRC. Metachronous liver metastasis was defined as liver metastasis occurring more than 6 months after radical resection of CRC (30).

### Blood sample analysis

Routine blood tests were performed to detect neutrophils and lymphocytes. The NLR was defined as the absolute count of neutrophils divided by the absolute count of lymphocytes. Markers of inflammation include neutrophil count, lymphocyte count, NLR, and LDH. Both CEA and CA 19–9 are tumor markers.

### Statistical analysis

Variables were described by categorical variables, and the clinical and pathological characteristics of patients were summarized by descriptive analysis. The optimal cut-off value of the NLR for the OS analysis was calculated using a receiver operating characteristic (ROC) curve. The chi-square test of categorical variables was used to compare the differences between groups. Logistic regression analysis was used to analyze the risk factors for the time frame of CRLM. The probability of OS was estimated by the Kaplan–Meier method in the univariate survival analysis, and the log-rank test was used for comparison. A Cox proportional hazard regression model was used to obtain the survival risk hazard ratio (HR) and 95% confidence interval (CI). Significant predictors in the univariate analysis were further estimated by the forward stepwise (likelihood ratio) selection of multivariate analysis so that only significant variables had HR and 95% CI values within the threshold. Finally, area under the ROC curve (AUC) was used to evaluate the performance of the model. The above statistical analyses were conducted using SPSS version 25.0, R version 3.6.1 and GraphPad Prism9, and differences with a *p* < 0.05 were defined as statistically significant.

## Results

### Cut-off values of inflammatory markers

**Figure 1:**
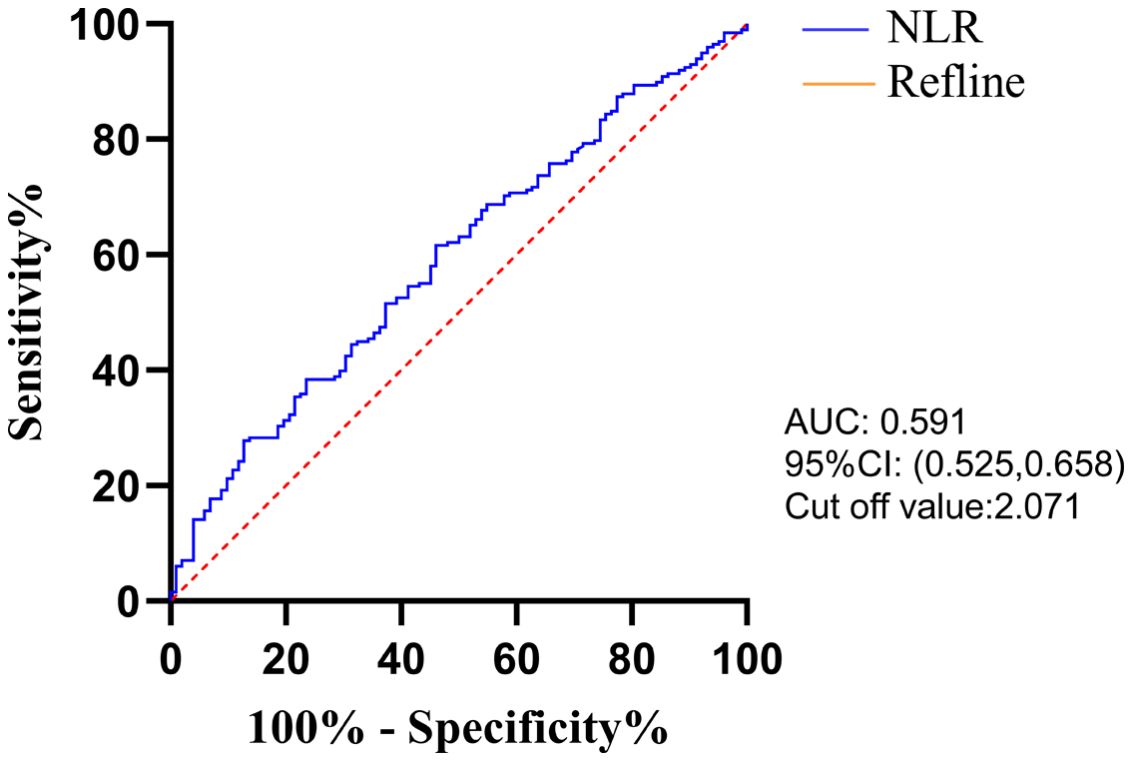
The ROC of preoperative NLR.

The optimal cut-off value for the NLR based on the time of liver metastasis was calculated by ROC curve analysis and was found to be 2.071 (AUC: 0.591, Figure 1). According to laboratory reference values, the normal ranges of CEA, CA19-9 and LDH are 0–5 ng/mL, 0–37 U/mL and 120–250 U/L, respectively. According to the NLR-LDH score, we categorized patients into three groups: high score [NLR ≤ 2.071 and LDH ≤ upper limit normal (ULN)], intermediate score (NLR > 2.071 or LDH > ULN) and low score (NLR > 2.071 and LDH > ULN).

### Baseline characteristics of patients

**Figure 2:**
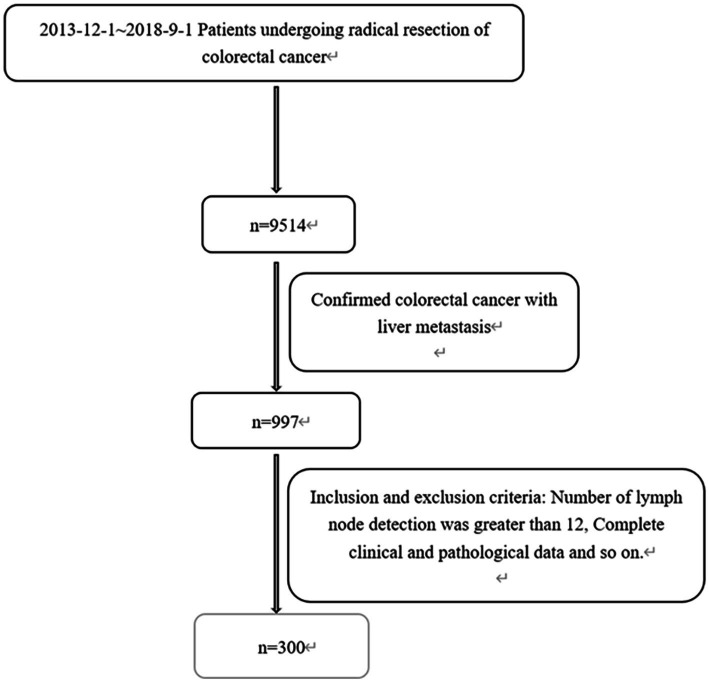
Flow diagram of patient disposition.

A total of 9,514 CRC patients were assessed and included, and 997 patients (10.5%) developed LM. Patients without key data on inflammatory markers, patients with infection or blood disease, and patients lost to follow-up were excluded. Finally, 300 patients with CRLM were included in the study (Figure 2). There were 197 males (65.7%) and 103 females (34.3%), with a median age of 60 years (23–91 years) at the time of diagnosis. The median survival time was 30 months. Overall, in the study cohort, according to the AJCC-TNM staging, there were 14 patients (4.6%) in stage I, 58 patients (19.0%) in stage II, 93 patients (30.5%) in stage III, and 124 patients (43.9%) in stage IV. During the study period, 152 patients died from cancer-related causes. Additional information, including clinical, pathological, and therapeutic characteristics, is summarized in Table 1.

**Table 1:** Baseline characteristics of the study population.

Variables		Frequencies, n (%) (*n* = 300)
Age (year)		
	>60	147 (49)
	≤60	153 (51)
Gender		
	Male	197 (65.7)
	Female	103 (34.3)
Sidedness		
	Left	128 (42.7)
	Right	49 (16.3)
	Rectum	123 (41.0)
Primary tumor size		
	T4	56 (18.7)
	T1-3	244 (81.3)
R0		
	Yes	162 (54.0)
	No	138 (46.0)
Histology		
	Well or moderately differentiated	265 (88.3)
	Por, Sig or Muc	34 (11.3)
	Missing	1 (0.3)
Metastatic sites		
	Only liver	231 (77.0)
	Others	69 (23.0)
Lymph node metastasis		
	Yes	196 (65.3)
	No	104 (34.7)
Preoperative therapy		
	Yes	58 (19.3)
	No	242 (80.7)
KRAS		
	Wild type	219 (73.0)
	Mutated	81 (27.0)
CEA (ng/mL)		
	≤5	133 (44.3)
	>5	166 (55.3)
	Missing	1 (0.3)
CA199(U/mL)		
	≤37	202 (67.3)
	>37	95 (31.7)
	Missing	3 (1.0)
LDH(U/L)		
	≤250	260 (86.7)
	>250	40 (13.3)
NLR		
	≤2.071	131 (43.0)
	>2.071	169 (55.4)

NLR, neutrophil-to-lymphocyte ratio; LDH, lactate dehydrogenase; Por, Poorly differentiated; Sig, Signet-cell; Muc, Mucinous; CEA, carcinoembryonic antigen; CA19-9, carbohydrate antigen 19–9.

The chi-square test showed significant differences in tumor location, R0 resection, NLR, CEA, CA19-9, LDH, NLR-LDH values, KRAS mutation with CRLM stage (*p* < 0.05) (Table 2).

**Table 2:** Baseline characteristics of patients.

Variables	Parameters	Liver metastasis time		*p*
		Synchronous LM (%)	Metachronous LM (%)	
Age (year)	>60	104 (52.5)	43 (42.2)	
	≤60	94 (47.5)	59 (57.8)	0.089
Gender	Male	129 (65.2)	68 (66.7)	
	Female	69 (34.8)	34 (33.3)	0.793
Primary tumor	Colon	131 (66.2)	46 (45.1)	
	Rectum	67 (33.8)	56 (54.9)	**0.001**
Histology	Poorly	20 (10.1)	14 (13.9)	
	Well	178 (89.9)	87 (86.1)	0.262
T classification	T4	43 (21.7)	13 (12.7)	
	T1-3	155 (78.3)	89 (87.3)	0.059
N classification	N1-2	134 (67.7)	62 (60.8)	
	N0	64 (32.3)	40 (39.2)	0.235
R0	Yes	72 (36.4)	90 (88.2)	
	No	126 (63.6)	12 (11.8)	**0.000**
NLR	>2.071	122 (61.6)	47 (46.1)	
	≤2.071	76 (38.4)	55 (53.9)	**0.010**
CEA (ng/mL)	>5	126 (63.6)	41 (40.2)	
	≤5	72 (36.4)	61 (59.8)	**0.000**
CA199 (U/mL)	>37	79 (39.9)	16 (15.7)	
	≤37	119 (60.1)	86 (84.3)	**0.000**
LDH (U/L)	>250	38 (19.2)	2 (2.0)	
	≤250	160 (80.8)	100 (98.0)	**0.000**
KRAS	Mutated	62 (31.3)	19 (18.6)	
	Wild type	136 (68.7)	83 (81.4)	**0.019**
NLR-LDH	High	32 (16.2)	1 (1.0)	
	Intermediate	96 (48.5)	47 (46.1)	
	Low	70 (35.4)	54 (52.9)	**0.000**

Por, Poorly differentiated; Sig, Signet-cell; Muc, Mucinous; NLR, neutrophil-to-lymphocyte ratio; LDH, lactate dehydrogenase; low [NLR ≤ 2.071 and LDH ≤ upper limit normal (ULN)], intermediate (NLR > 2.071 or LDH > ULN) and high (NLR > 2.071 and LDH > ULN); CEA, carcinoembryonic antigen; CA19-9, carbohydrate antigen 19–9.

Bold values are statistically significant.

The clinicopathological features grouped by NLR, LDH and NLR-LDH values are shown in Table 3. The values for NLR, LDH and NLR-LDH were significantly correlated with the LM time of CRC (*p* < 0.05). There were significantly more cases of synchronous LM in the high NLR group (*p* = 0.010), the high LDH group (*p* < 0.001) and the high NLR-LDH group (*p* < 0.001). NLR, LDH and NLR-LDH were also significantly correlated with age (*p* < 0.050).

**Table 3:** Correlations between clinical characteristics.

Variables	Parameters	NLR		*p*	LDH		*p*	NLR-LDH			*p*
		>2.071 (%)	≤2.071 (%)		≤250 (%)	>250 (%)		Low (%)	Intermediate (%)	High (%)	
Age (year)	>60	96 (56.8)	51 (38.9)		12 (46.5)	26 (65.0)		47 (37.9)	78 (54.5)	22 (66.7)	
	≤60	73 (43.2)	80 (61.1)	**0.002**	139 (53.5)	14 (35.0)	**0.030**	77 (62.1)	65 (45.5)	**11 (33.3)**	**0.002**
Gender	Male	117 (69.2)	80 (61.1)		172 (66.2)	25 (62.5)		75 (60.5)	102 (71.3)	20 (60.6)	
	Female	52 (30.8)	51 (38.9)	0.140	88 (33.8)	15 (37.5)	0.650	49 (39.5)	41 (28.8)	13 (39.4)	0.143
Primary tumor	Colon	111 (65.7)	65 (50.0)		151 (58.3)	25 (62.5)		63 (51.2)	90 (62.9)	23 (69.7)	
	Rectum	58 (34.3)	65 (50.0)	**0.006**	108 (41.7)	15 (37.5)	0.615	60 (48.8)	53 (37.1)	10 (30.3)	0.062
Histology	Well	19 (11.3)	15 (11.5)		227 (87.6)	38 (95.0)		15 (12.1)	17 (12.0)	2 (6.1)	
	Poorly	149 (88.7)	116 (88.5)	0.970	32 (12.4)	2 (5.0)	0.173	109 (87.9)	125 (88.0)	31 (93.9)	0.595
T classification	T4	35 (20.7)	21 (16.0)		48 (18.5)	8 (20.0)		20 (16.1)	29 (20.3)	7 (21.2)	
	T1-3	134 (79.3)	110 (84.0)	0.302	212 (81.5)	32 (80)	0.816	104 (83.9)	114 (79.7)	26 (78.8)	0.634
N classification	N1-2	114 (67.5)	82 (62.6)		161 (61.9)	35 (87.5)		78 (62.9)	87 (60.8)	31 (93.9)	
	N0	55 (32.5)	49 (37.4)	0.380	99 (38.1)	5 (12.5)	**0.002**	46 (37.1)	56 (39.2)	2 (6.1)	**0.001**
Liver metastases	Synchronous	122 (72.2)	76 (58.0)		160 (61.5)	38 (95.0)		70 (56.5)	96 (67.1)	32 (97.0)	
	Metachronous	47 (27.8)	55 (42.0)	**0.010**	100 (38.5)	2 (5.0)	**0.000**	54 (43.5)	47 (32.9)	1 (3.0)	**0.000**
The number of LM	>3	63 (37.5)	42 (32.3)		179 (68.4)	14 (35.0)		38 (30.9)	45 (31.7)	22 (66.7)	
	≤3	105 (62.5)	88 (67.7)	0.352	79 (30.6)	26 (65.0)	**0.000**	85 (69.1)	97 (68.3)	11 (33.3)	**0.000**
R0	Yes	83 (49.1)	79 (60.3)		154 (59.2)	8 (20.0)		77 (62.1)	79 (55.2)	6 (18.2)	
	No	86 (50.9)	52 (39.7)	0.054	106 (40.8)	32 (80.0)	**0.000**	47 (37.9)	64 (44.8)	27 (81.8)	**0.000**
Other metastases	Yes	39 (23.1)	30 (22.9)		197 (75.8)	34 (85)		28 (22.6)	37 (25.9)	4 (12.1)	
	No	130 (76.9)	101 (77.1)	0.971	63 (24.2)	6 (15)	0.197	96 (77.4)	106 (74.1)	29 (87.9)	0.236
Preoperative therapy	Yes	24 (14.2)	34 (26.0)		53 (20.4)	5 (12.5)		32 (25.8)	23 (16.1)	3 (9.1)	
	No	145 (85.8)	97 (74.0)	**0.011**	207 (79.6)	35 (87.5)	0.240	92 (74.2)	120 (83.9)	30 (90.9)	**0.038**

Well, Well or moderately differentiated; Poorly, Poorly differentiated, Signet-cell and Mucinous; LM, Liver metastases.

Bold values are statistically significant.

### Univariate analysis of inflammatory markers and clinicopathologic factors

Table 4 shows the univariate analysis of synchronous CRLM. In the analysis, a high NLR indicated a high likelihood of synchronous CRLM (HR: 1.878, 95% CI: 1.159–3.049, *p* = 0.011), Patients with elevated LDH had a much higher incidence of synchronous CRLM (HR: 11.875, 95% CI: 2.803–50.306, *p* = 0.001). In addition, the NLR-LDH value showed significant prognostic effects on synchronous CRLM, especially high NLR-LDH score (synchronous CRLM, *p* = 0.002, HR = 24.868, 95% CI: 3.269–186.417), although the intermediate group failed to have significant prognostic value. Tumors located in the rectum (HR = 2.362, 95% CI: 1.449–3.851, *p* = 0.001) and with CEA > 5 (HR = 2.583, 95% CI: 1.581–4.219, *p* < 0.001), CA19-9 > 37 (HR = 3.587, 95% CI: 1.958–6.573, *p* < 0.001) and mutant KRAS (HR: 1.991, 95% CI: 1.113–3.564, *p* = 0.020) were more likely to have synchronous LM than tumors with other features.

**Table 4:** Univariate logistic regression analysis of factors associated with liver metastases.

Variables	Parameters	Simultaneous LM	
		HR (95%Cl)	*P*
Age (year)	≤60	Reference	
	>60	1.518 (0.938–2.458)	0.090
Gender	Male	Reference	
	Female	1.070 (0.646–1.772)	0.793
Primary tumor	Rectum	Reference	
	Colon	2.362 (1.449–3.851)	**0.001**
Histology	Poorly	Reference	
	Well	1.432 (0.691–2.970)	0.335
T classification	T1-3	Reference	
	T4	1.899 (0.969–3.722)	0.062
N classification	N0	Reference	
	N1-2	1.351 (0.822–2.220)	0.235
NLR	≤2.071	Reference	
	>2.071	1.878 (1.159–3.046)	**0.011**
CEA (ng/mL)	≤5	Reference	
	>5	2.583 (1.581–4.219)	**0.000**
CA199 (U/mL)	≤37	Reference	
	>37	3.587 (1.958–6.573)	**0.000**
LDH (U/L)	≤250	Reference	
	>250	11.875 (2.803–50.306)	**0.001**
KRAS	Wild type	Reference	
	Mutated	1.991 (1.113–3.564)	**0.020**
NLR-LDH	Low	Reference	
	Intermediate	1.576 (0.958–2.592)	**0.073**
	High	24.686 (3.269–186.417)	**0.002**

Well,Well or moderately differentiated; Poorly, Poorly differentiated, Signet-cell and Mucinous; NLR, neutrophil-to-lymphocyte ratio; LDH, lactate dehydrogenase; low (NLR ≤ 2.071 and LDH ≤ 250 U/L), intermediate (NLR > 2.071 or LDH > 250 U/L) and high (NLR > 2.071 and LDH > 250 U/L); CEA, carcinoembryonic antigen; CA19-9, carbohydrate antigen 19–9.

Bold values are statistically significant.

In terms of OS (Table 5), patients with a high NLR showed significantly worse OS (HR: 1.583, 95% CI: 1.136–2.205, *p* = 0.007) than those with a low NLR, and the same was true of LDH (HR: 2.932, 95 CI: 1.970–4.365, *p* < 0.001). In addition, the NLR-LDH value showed significant prognostic effects on OS, especially for patients with high NLR-LDH scores (high, HR = 3.672, 95% CI: 2.262–5.960, *p* < 0.05). Similar trend was also observed for patients with low or intermediate NLR-LDH scores (intermediate, HR = 1.507, 95% CI: 1.053–2.157, *p* < 0.05), Moreover, our results indicated that age (*p* = 0.005), histology (*p* = 0.037), liver metastases (*p* = 0.003), CRLM number (*p* = 0.027), R0 (*p* = 0.024), preoperative therapy (*p* = 0.019), CEA (*p* = 0.006),CA19-9 (*p* < 0.001), T classification (<0.001), N classification (<0.001), and KRAS status (*p* = 0.048) were statistically significant.

**Table 5:** Univariate analyses for OS.

Factors		Univariate	
		HR (95%Cl)	*P*
Age	>60 vs. ≤ 60	1.582 (1.147–2.183)	**0.005**
Gender	Male vs. Female	1.164 (0.828–1.636)	0.383
Histology	Poorly vs. Well	1.637 (1.030–2.602)	**0.037**
Primary tumor location	Colon vs. Rectum	1.199 (0.864–1.662)	0.275
Liver metastases	Simultaneous vs. Heterochronous	1.720 (1.198–2.469)	**0.003**
Other metastasis	Yes vs. No	1.184 (0.816–1.718)	0.375
Number of metastasis	>3 vs. ≤3	1.446 (1.043–2.066)	**0.027**
R0	Yes vs. No	0.570 (0.413–0.785)	**0.001**
Preoperative therapy	Yes vs. No	0.734 (0.481–1.121)	**0.019**
CEA (ng/mL)	>5 vs. ≤5	1.587 (1.142–2.207)	**0.006**
CA199 (U/mL)	>37 vs. ≤37	1.881 (1.357–2.607)	**0.000**
T classification	T4 vs. T1-3	2.194 (1.515–3.176)	**0.000**
N classification	N1-2 vs. N0	2.388 (1.629–3.500)	**0.000**
KRAS	Yes vs. No	1.420 (1.004–2.010)	**0.048**
NLR	>2.071 vs. ≤2.071	1.583 (1.136–2.205)	**0.007**
LDH (U/L)	>250 vs. ≤250	2.932 (1.970–4.365)	**0.000**
NLR + LDH	Intermediate vs. low	1.507 (1.053–2.157)	**0.025**
	High vs. low	3.672 (2.262–5.960)	**0.000**

NLR, neutrophil-to-lymphocyte ratio; LDH, lactate dehydrogenase; low (NLR ≤ 2.071 and LDH ≤ 250 U/L), intermediate (NLR > 2.071 or LDH > 250 U/L) and high (NLR > 2.071 and LDH > 250 U/L); CEA, carcinoembryonic antigen; CA19-9, carbohydrate antigen 19–9.

Bold values are statistically significant.

**Figure 3:**
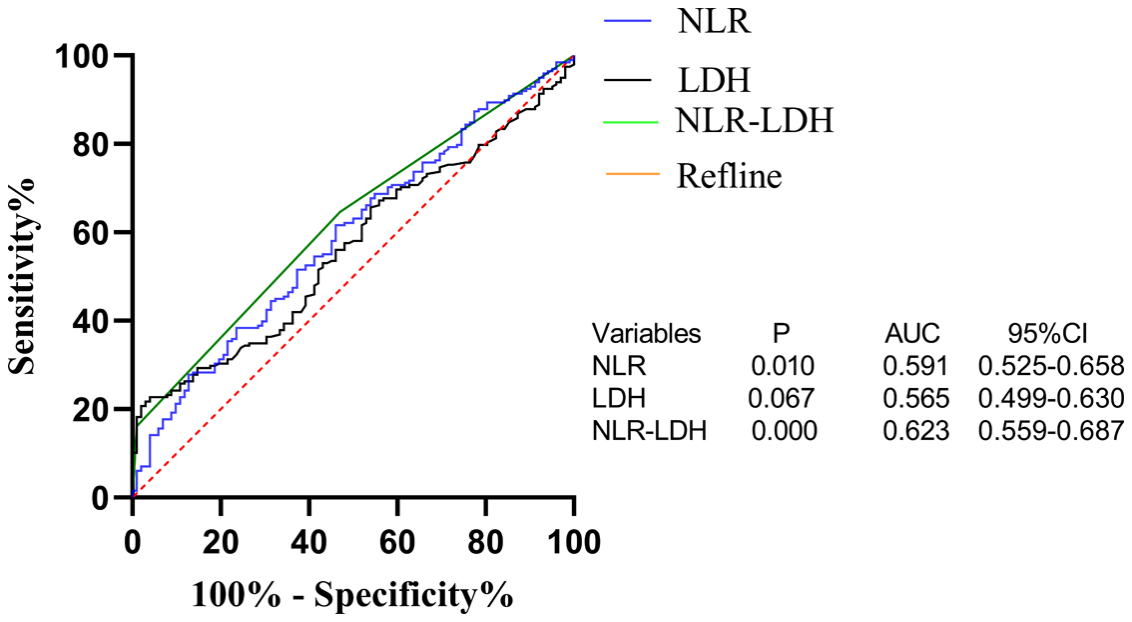
ROC curve of preoperative peripheral blood NLR, LDH and NLR-LDH combined in patients for predicting liver metastasis time of colorectal cancer.

The results of Kaplan–Meier survival curves are shown in Figure 3. The combination of a high NLR and elevated LDH and NLR-LDH levels suggested a poor prognosis and a significantly shorter median survival time.

### Multivariate analysis of inflammatory markers

**Figure 4:**
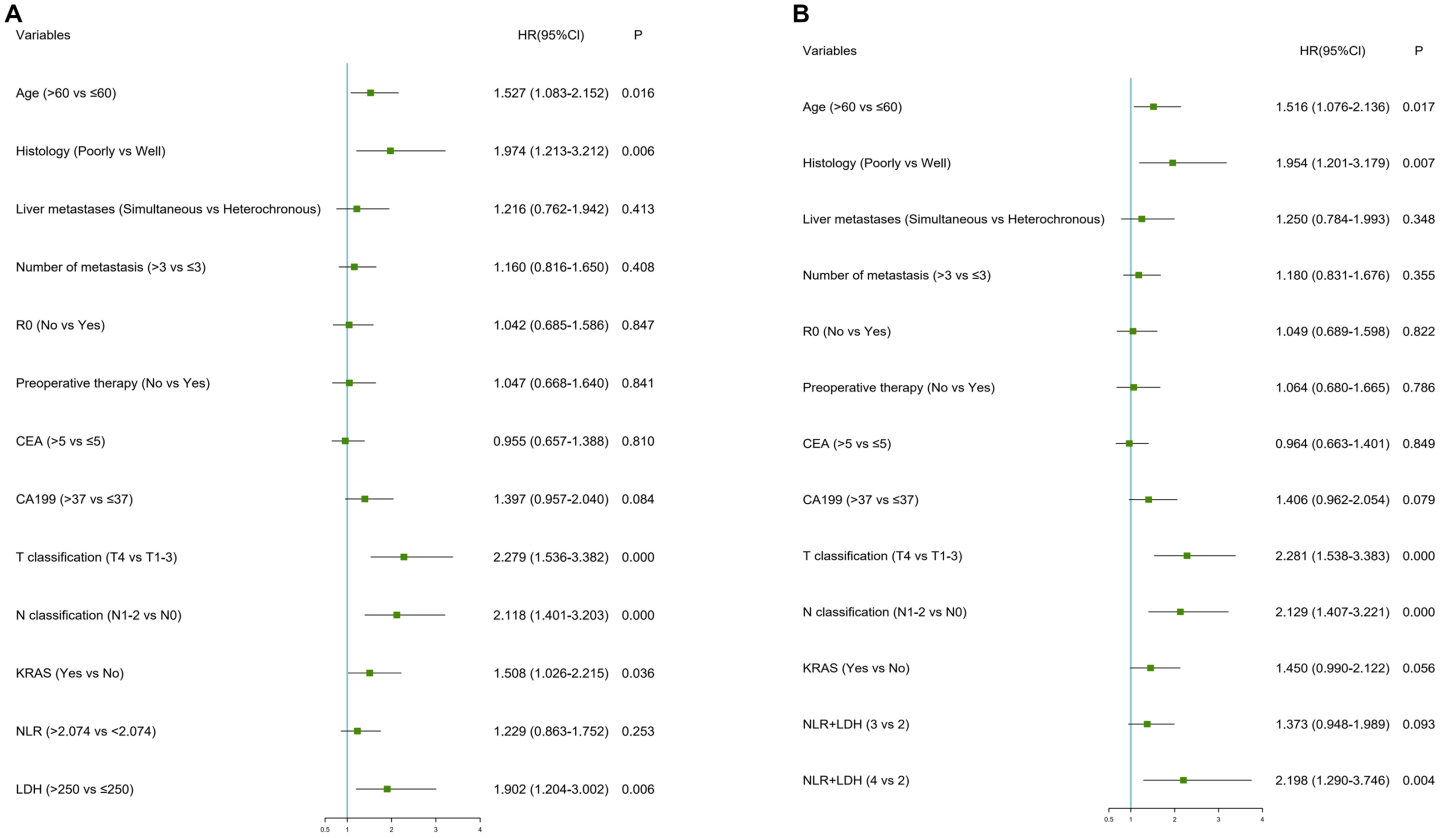
Forest plots of hazard ratios according to multivariate analyses for OS.

In terms of synchronous CRLM, KRAS status and CEA, LDH and NLR-LDH values were found to be statistically significant, with independent predictive value (Tables 6, 7).Our results indicated that T stage (*p* < 0.001), N stage (*p* < 0.001) and poor histology (*p* < 0.05) were independently associated with poor OS (forest plots, Figure 4). There was a significant difference in OS between the elevated and normal LDH groups (*p* < 0.05), although the limited number of patients with NLR > 2.071 affected the validity of the results. A high NLR-LDH was an independent predictor of OS (HR = 2.198, 95% Cl: 1.290–3.746, *p* = 0.004). T and N stage were significant influencing factors for survival and CRLM in clinical practice, so even though no statistical significance was found in the single-factor analysis, they were still included in the multi-factor analysis (forest plots, Figure 4).

**Table 6:** Multivariate logistic analysis of factors associated with liver metastases.

Variables	Parameters	Synchronous LM	
		HR (95%Cl)	*P*
Primary tumor	Rectum	Reference	
	Colon	1.709 (0.990–2.952)	0.055
T classification	T1-3	Reference	
	T4	1.395 (0.666–2.924)	0.378
N classification	N0	Reference	
	N1-2	0.892 (0.508–1.564)	0.689
KRAS	Wild type	Reference	
	Mutated	1.911 (1.017–3.589)	**0.044**
CEA (ng/mL)	≤5	Reference	
	>5	1.775 (1.016–3.099)	**0.044**
CA199 (U/mL)	≤37	Reference	
	>37	2.236 (1.149–4.353)	**0.018**
NLR	≤2.071	Reference	
	>2.071	1.500 (0.879–2.557)	0.137
LDH (U/L)	≤250	Reference	
	>250	9.040 (2.065–39.567)	**0.003**

NLR, neutrophil-to-lymphocyte ratio; LDH, lactate dehydrogenase; CEA, carcinoembryonic antigen; CA19-9, carbohydrate antigen 19–9.

Bold values are statistically significant.

**Table 7:** Multivariate logistic analysis of factors associated with liver metastases.

Variables	Parameters	Synchronous LM	
		HR (95%Cl)	*P*
Primary tumor	Rectum	Reference	
	Colon	1.633 (0.950–2.807)	0.076
T classification	T1-3	Reference	
	T4	1.397 (0.668–2.923)	0.375
N classification	N0	Reference	
	N1-2	0.877 (0.501–1.534)	0.645
KRAS	Wild type	Reference	
	Mutated	1.881 (1.003–3.531)	**0.049**
CEA (ng/mL)	≤5	Reference	
	>5	1.822 (1.047–3.172)	**0.034**
CA199 (U/mL)	≤37	Reference	
	>37	2.238 (1.152–4.347)	**0.017**
NLR-LDH	Low	Reference	
	Intermediate	1.551 (0.910–2.645)	0.107
	High	18.470 (2.388–142.843)	**0.005**

NLR, neutrophil-to-lymphocyte ratio; LDH, lactate dehydrogenase; low (NLR ≤ 2.071 and LDH ≤ 250 U/L), intermediate (NLR > 2.071 or LDH > 250 U/L) and high (NLR > 2.071 and LDH > 250 U/L); CEA, carcinoembryonic antigen; CA19-9, carbohydrate antigen 19–9.

Bold values are statistically significant.

### ROC curve analysis for comparison of the predictive ability of NLR, LDH, and NLR-LDH combined

**Figure 5:**
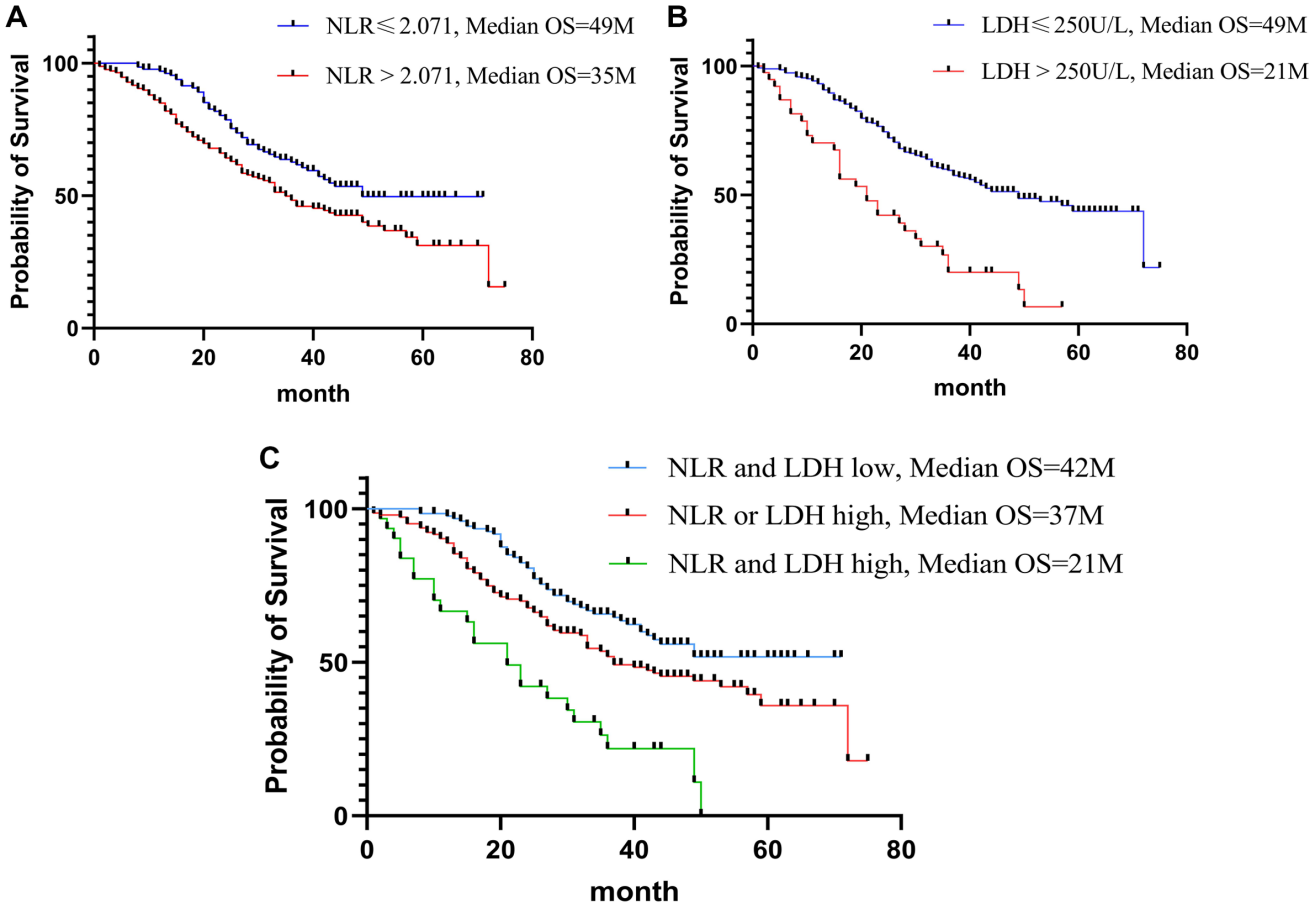
Kaplan-Meier curves of OS for NLR **(A)**; Kaplan-Meier curves of OS for LDH **(B)**; Kaplan-Meier curves of OS for NLR-LDH **(C)**.

**Figure 6:**
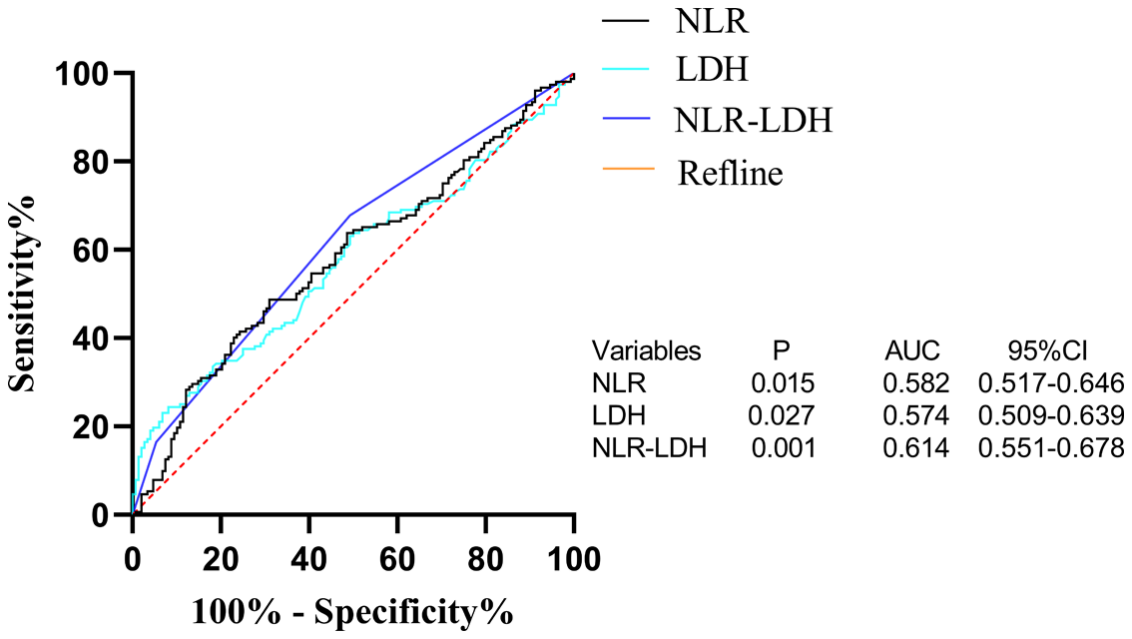
ROC curve of preoperative peripheral blood NLR, LDH and NLR-LDH combined in patients for predicting OS.

The NLR-LDH score has better predictive ability than the NLR or LDH score. The ROC curve analysis showed that the predictive value of the NLR-LDH score for synchronous CRLM (*p* = 0.010) and OS (*p* = 0.001) was superior to that of the NLR or LDH score alone (Figure 5). In the NLR-LDH scoring model, the AUC of synchronous CRLM was predicted to be 0.623 ± 0.033, while the AUC of OS was predicted to be 0.614 ± 0.032 (Figure 6).

## Discussion

Accumulating studies have shown that different types of systemic inflammatory cytokines can be used as prognostic markers to determine the prognosis of human malignant tumors (31). Inflammation is reported to play an important role in the multistage progression of tumorigenesis and is now considered a marker of cancer (6). Chronic inflammation can cause genotoxic stress, induce tumor cell proliferation, increase angiogenesis and tissue infiltration, and facilitate tumor proliferation (8). The tumor immunosuppressive state can help the tumor evade immune surveillance and promote tumor proliferation and progression (24). The peripheral blood indexes NLR and LDH, whose evaluation methods are convenient and non-invasive, are gradually becoming important markers to judge the prognosis of tumor patients and have great research potential and value (32). In our study, a ROC curve was used to redefine the cut-off value of the NLR. In addition, the timing of CRLM and prognosis were analyzed using NLR, LDH, and NLR-LDH values.

### NLR, LDH, and the synchronous LM of CRC

The liver is the most common metastatic site of CRC. CRLM occurs in 15–25% of patients with CRC at the first diagnosis, while CRLM occurs in another 50% of patients during the progression of CRC (33). The treatment and prognosis of CRC with CRLM are significantly different from that of CRC without CRLM. Some CRCs already have minor CRLM that cannot be detected by imaging. Therefore, it is very important to identify whether CRC has CRLM and conduct intervention in the early stage. In our study, we proposed for the first time the use of NLR, LDH, and NLR-LDH indicators to predict the timing of CRLM and conducted univariate and multivariate analyses of risk factors for synchronous CRLM. The results confirmed that increases in NLR, LDH, and NLR-LDH indexes were all risk factors for the concurrent CRLM, and the LDH and NLR-LDH indexes were independent risk factors.

The NLR in peripheral blood has been widely studied as an important indicator of systemic inflammation in recent years (14, 34). Its elevation indicates a relative increase in neutrophils and a decrease in lymphocytes, indicating a high degree of systemic inflammation. The persistence of the systemic inflammatory response can regulate the tumor microenvironment and promote tumor progression and metastasis. As shown in our study, an elevated NLR in CRC patients indicates the presence of a systemic inflammatory response, and a higher NLR (NLR > 2.071) is associated with a higher risk of concurrent CRLM.

According to previous studies, elevated LDH subtypes are more common in malignant tumors than in normal cells (35). In malignant tumors, tumor cells are more likely to obtain energy through glycolysis in the presence of oxygen, and LDH is a key enzyme in glycolysis. The glycolytic metabolite lactic acid can regulate cellular immune metabolism in the tumor microenvironment, promote immunosuppression in the tumor region, and facilitate tumor progression and metastasis (36). Our study confirmed that LDH is an independent risk factor of synchronous CRLM and an important indicator for the diagnosis of CRLM.

We found that the preoperative peripheral blood NLR-LDH score had a higher AUC for the diagnosis of concurrent CRLM than either of the independent diagnostic indexes (NLR + LDH_AUC_ = 0.623, NLR_AUC_ = 0.591, LDH_AUC_ = 0.565). The reason may be that this value can reflect the inflammatory response and immune function of patients with CRC. The combination of NLR and LDH can make up for the limitations of a single-indicator diagnosis and increase the diagnostic accuracy to some extent. In clinical practice, rational use of the preoperative peripheral blood NLR-LDH index combined with traditional imaging examination can more accurately predict whether patients with CRC have concurrent CRLM, providing justification for necessary further treatment.

Our study also found that colon cancer is more likely to develop concurrent CRLM than rectal cancer, possibly because colon cancer tumor cells are more likely to return to the portal vein through the inferior mesenteric vein, thus promoting the occurrence of the concurrent CRLM. KRAS mutation in CRC indicates a poor prognosis (37). In our study, it was found that CRC patients with KRAS mutations are more likely to have concurrent CRLM than patients without KRAS mutations and that KRAS mutation status is as an independent risk factor. Moreover, increased preoperative CEA and CA19-9 are also risk factors for concurrent CRLM, which is consistent with the findings of Zheng et al. (38).

### NLR, LDH, and the OS of CRC

In recent years, NLR and LDH have been identified to be associated with the survival and prognosis of patients with a variety of malignant tumors, including lung cancer (39), esophageal cancer (40), melanoma (41), and uterine sarcoma (42). As an indicator reflective of neutrophil and lymphocyte counts, the NLR may reflect systemic inflammatory responses (43). Neutrophils can be categorized as N1 and N2 phenotype by TGF-beta signaling in the tumor microenvironment (44). N2 neutrophils can produce vascular endothelial growth factor (VEGF) and metalloproteinase, promoting tumor angiogenesis, invasion and metastasis (45), while lymphocytes play an important role in antitumor immunity (46). Consistent with our findings, an elevated NLR is a risk factor influencing prognosis in patients with CRLM. As shown in the survival curve (Figure 3), the OS of patients with CRLM and an elevated NLR was lower than that of those with a decreased NLR (median OS: 35 vs. 49 months).

Increased LDH promotes tumor progression by regulating tumor metabolism and the microenvironment and serves as an indicator of poor prognosis in cancer patients (35, 47). Our study also found that LDH is an independent risk factor for the prognosis of CRLM patients. As shown in the survival curve, the OS of CRLM patients and elevated LDH was lower than that of those with decreased LDH (median OS: 21 vs. 49 months).

More importantly, when the NLR and LDH indexes were combined to evaluate the prognosis of CRLM, patients in the high NLR-LDH group had significantly lower OS than those in either the intermediate group or the low group (median OS: 21 vs. 37 vs. 42 months), and this index was an independent risk factor. A simultaneous increase in NLR and LDH means that the body is in a state of systemic inflammatory response and immunosuppression, which makes the tumor microenvironment more suitable the growth and metastasis of tumor cells, leading to a poor prognosis. The preoperative detection of both NLR and LDH provides a great reference for the assessment of the prognosis of CRLM patients.

In our study, independent risk factors for CRLM included age, pathological characteristics, T and N stage, and KRAS mutation status. As patient age over 60 years old, the OS decreased. When the pathological type of CRC was unfavorable and the stage of the primary tumor was late, the tumor was more malignant and the OS was shorter.

## Conclusion

Overall, in this study we used the NLR, LDH, and NLR-LDH values to predict the time of CRLM and the OS in CRC. We propose for the first time a prognostic risk scoring model based on the NLR and LDH level to predict the risk of concurrent CRLM and the prognosis of CRC patients. LDH and NLR-LDH are reliable, independent laboratory biomarkers that can be easily obtained in the clinic to predict synchronous CRLM and OS in CRC patients. NLR, LDH, and NLR-LDH may help to guide the use of therapeutic strategies and cancer surveillance.

## Data availability statement

The original contributions presented in the study are included in the article/supplementary material, further inquiries can be directed to the corresponding authors.

## Ethics statement

The studies involving human participants were reviewed and approved by the full name: the role of LDH expression regulation in liver metabolic reprogramming in colorectal cancer with liver metastasis. The patients/participants provided their written informed consent to participate in this study. Written informed consent was obtained from the individual(s) for the publication of any potentially identifiable images or data included in this article.

## Author contributions

QC designed and conceived the study. QC, Z-PC, and J-YW collected and analyzed the data. QC and J-DY performed the statistical analysis. QC and Z-YL drafted the manuscript. Y-LW, G-lL, and Z-YL critically revised all the intellectual content of the manuscript. All authors have read and approved the final manuscript.

## Funding

This work was supported by the Science and Technology Plan Foundation of Guangzhou (202201010954).

## Conflict of interest

The authors declare that the research was conducted in the absence of any commercial or financial relationships that could be construed as a potential conflict of interest.

## Publisher’s note

All claims expressed in this article are solely those of the authors and do not necessarily represent those of their affiliated organizations, or those of the publisher, the editors and the reviewers. Any product that may be evaluated in this article, or claim that may be made by its manufacturer, is not guaranteed or endorsed by the publisher.
